# Leriche’s syndrome and twin pregnancy

**DOI:** 10.4274/tjod.galenos.2019.58219

**Published:** 2020-04-06

**Authors:** Cemal Tamer Erel, Hakan Erenel, Ayşegül Mut, Kılıç Aydınlı

**Affiliations:** 1 İstanbul University-Cerrahpaşa, Cerrahpaşa Faculty of Medicine, Department of Obstetrics and Gynecology, İstanbul, Turkey; 2 Medicus Health Center, Department of Obstetrics and Gynecology, İstanbul, Turkey

**Keywords:** Aortoiliac occlusion, Leriche’s syndrome, pregnancy

## Abstract

Leriche’s syndrome is characterized by chronic obstruction of the abdominal aorta and iliac arteries. A patient with Leriche’s syndrome presented with twin pregnancy and severe preeclampsia at 32 weeks’ gestation. Cesarean delivery was performed and the patient was admitted to the intensive care unit. Magnetic resonance angiography showed total occlusion of the distal abdominal aorta, common, and external iliac arteries. There were extensive collateral vessels between the lumbar arteries and iliolumbar arteries. The patient was discharged in an improved clinical condition. Leriche’s syndrome and pregnancy demonstrating complete aortic, common, and external iliac artery occlusion is very rare in the literature. Despite complete occlusion, viability of the fetus can be achieved with collateral vessels.

## Introduction

Leriche syndrome, also known as aortoiliac occlusive disease, is characterized by chronic obstruction of the abdominal aorta and iliac arteries^(1)^. The disease was first described by Robert Graham in 1814^(2)^. Leriche syndrome was named after a French surgeon, Rene Leriche, who first operated on the condition^(1)^. We aimed to report a case of Leriche syndrome and twin pregnancy presenting with severe preeclampsia because there are scant data regarding total aortoiliac occlusion and pregnancy in the literature.

## Case report

**Figure 1:**
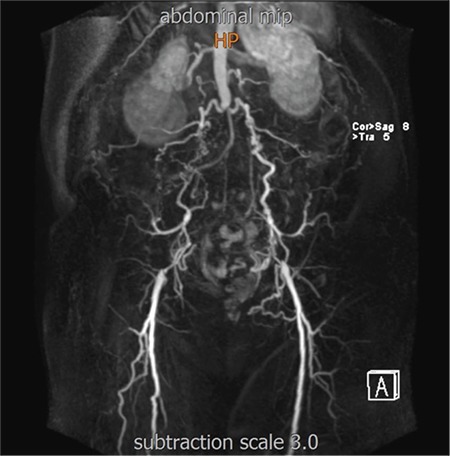
Magnetic resonance angiography showed total occlusion of distal abdominal aorta. Common iliac and external iliac vessels were bilaterally occluded. Extensive collateral vessels were seen between the lumbar arteries and iliolumbar arteries

A 53-year-old gravida-1-para-0 at 32 weeks’ gestation was referred to hospital for dyspnea and uncontrolled hypertension. Her medical history revealed chronic hypertension, pregestational diabetes mellitus (DM), and aortoiliac occlusive disease. She had a twin pregnancy using egg donation in another country. She was treated with a regimen of alpha methyl dopa (3x250 mg), acetylsalicylic acid (1x100 mg), and enoxaparin (1x6000 IU). Her antenatal visits were irregular. In his initial physical examination blood pressure was 190/90 mm Hg. A fetal ultrasound examination showed a dichorionic diamniotic twin pregnancy. Hematologic and serum biochemical tests were within normal limits. Urine dipstick analysis revealed 3+ proteinuria. Elevated blood pressure persisted despite antihypertensive drugs such as calcium channel blockers and a diagnosis of superimposed preeclampsia was made. A decision for immediate delivery was made. Cesarean section was performed due to a breech cephalic presentation. A male infant weighing 1.730 g and female infant weighing 1.980 g were delivered. The patient was admitted to the intensive care unit due to a history of aortoiliac occlusive disease and severe hypertension. The postoperative period was uneventful and aortofemoropopliteal magnetic resonance (MR) angiography was performed at postoperative day 5. MR angiography showed total occlusion of the distal abdominal aorta. Common iliac and external iliac vessels were bilaterally occluded. Extensive collateral vessels were seen between the lumbar arteries and iliolumbar arteries (Figure 1). The patient was discharged on postoperative day 6. The infants were discharged in good condition. Informed consent was obtained.

## Discussion

Leriche syndrome is a rare atherosclerotic occlusive disease characterized by total occlusion in the abdominal aorta and/or both iliac arteries^(1)^. Aortoiliac lesions were categorized by the Trans-atlantic Inter-Society Consensus for the Management of Peripheral Arterial Disease (TASC II), and Leriche syndrome can be categorized as a type D aortoiliac lesion^(3)^. It has been reported that Leriche syndrome usually affects men in the third to sixth decades of life^(4)^. Hyperlipidemia, hypertension, DM, and smoking are described as risk factors in the literature^(5)^. Oocyte donation, maternal age, chronic hypertension, and pregestational DM were possible risk factors in our case. A previous study of 77 patients with aortoiliac disease showed increased pregnancy loss compared with the control group. In this study, a stenotic lesion of >50% was accepted for peripheral arterial occlusive disease^(6)^. Sass et al.^(7)^ reported a case of pregnancy with an aortic prosthesis for Leriche syndrome. In this case, the patient had previously undergone surgery and the abdominal aorta was the only affected aortic area. In comparison with these reports, there was total occlusion of the abdominal aorta, common, and external iliac arteries in our case. Patients with aortoiliac occlusive disease can be asymptomatic or present with several symptoms such as claudication, severe pain during rest, paresis, and absence of femoral pulses, which are highly dependent on the location of the occlusive lesion and development of collateral vessels^(8)^. We observed extensive collateral vessels in the distal region of the internal iliac artery. We can argue that these anastomoses between the lumbar arteries and iliolumbar arteries maintained normal uterine perfusion and the asymptomatic presentation of our patient can be explained also by impressive formation of retroperitoneal anastomoses. It is difficult to state an optimal obstetric management and follow-up for asymptomatic cases with aortoiliac occlusive disease because there are only few reports regarding aortoiliac occlusive disease and pregnancy in the literature.

We reported twin pregnancy and Leriche syndrome with complete aortic, common, and external iliac artery occlusion. Despite complete occlusion, viability of a fetus can be achieved with collateral vessels. Multidisciplinary teams must be involved in the care of these women.
